# Role of Impaired Oxalate Homeostasis in Cardiovascular Disease in Patients With End-Stage Renal Disease: An Opinion Article

**DOI:** 10.3389/fphar.2021.692429

**Published:** 2021-05-28

**Authors:** Natalia Stepanova

**Affiliations:** State Institution “Institute of Nephrology National Academy of Medical Science of Ukraine”, Kyiv, Ukraine

**Keywords:** oxalate homeostasis, cardiovascular disease, end-stage renal disease, oxidative stress, inflammation

## Introduction

Oxalate is an ionic form of a potentially toxic oxalic acid that is formed in the human body from a combination of food sources and their absorption in the gastrointestinal tract (approximately 30%), as well as the endogenous synthesis of glyoxylate (40%) and ascorbic acid (30%) (Jonassen et al., 2005; Robijn et al., 2011). The balance of oxalate in the human body is achieved due to its renal (up to 90%) and intestinal (10%) excretion (Robijn et al., 2011; Huang et al., 2020). Loss of kidney function decreases renal oxalate clearance and increases plasma oxalic acid (POx) concentration, according to the progression of chronic kidney disease (CKD) stages (Robijn et al., 2011; Perinpam et al., 2017). The accumulation of oxalate may be associated with oxidative stress, inflammation (Khan, 2014; Ermer et al., 2016; Dominguez-Gutierrez et al., 2018; Korol et al., 2021) and a high risk of cardiovascular disease (CVD) (Liu et al., 2014; Fan X et al., 2017; Arafa et al., 2020; Demikhov et al., 2020) in patients with kidney stones. However, although impaired oxalate homeostasis is a well-known occurrence in patients with end-stage renal disease (ESRD), a high POx concentration has never been considered a trigger for oxidative stress, systemic inflammation, and CVD risk in these patients. In this opinion article, based on the published data and results of our clinical studies, we outlined the possible contribution of oxalate to oxidative stress, chronic inflammation, and CVD risk in patients with ESRD.

### Gut Microbiota Disruption Causes Hyperoxalemia in Patients With ESRD

Given that oxalate is mainly excreted by the kidneys, dialysis seems to be the main approach for oxalate removal in patients with ESRD (Ermer et al., 2017; Perinpam et al., 2017). Nevertheless, despite a homogeneous patient population and a standardized dialysis regimen, the intraindividual predialysis POx concentration level varies from 1.8 mg/L (20 µmol/L) to 5.4 mg/L (60 µmol/L) in different studies (Ermer et al., 2017; Perinpam et al., 2017; Korol et al., 2021). Considering the marginal dependence of oxalate homeostasis on its dietary intake (Mitchell et al., 2019; Kumar et al., 2021) and limited renal excretion in patients with ESRD, it remains unclear why they have significant differences in POx concentration under the same treatment conditions. We hypothesized that in patients with anuria/kidney failure, the gut plays a much more significant role in oxalate handling than in healthy participants. Both paracellular and transcellular intestinal oxalate transport disruption and less functional activity of oxalate-degrading bacteria (ODB) in fecal microbiota might be the main factors affecting oxalate homeostasis in patients on dialysis.

The gastrointestinal tract plays a complex role in oxalate metabolism via intestinal oxalate transport and the ability of ODB to degrade oxalate (Hatch and Freel, 2008; Robijn et al., 2011; Huang et al., 2020; Ticinesi et al., 2020). Oxalate is absorbed from all parts of the gastrointestinal tract through paracellular (predominantly passive) and active transcellular mechanisms (Robijn et al., 2011; Ticinesi et al., 2020). The relative contribution of these two transport mechanisms varies with the intestinal segment and its condition (Hatch and Freel, 2008; Robijn et al., 2011; Whittamore and Hatch, 2017). Paracellular transport depends on the residence time of the chyme in the small intestine and the degree of calcium ionization (Hatch and Freel, 2008; Robijn et al., 2011; Whittamore and Hatch, 2017). The active transcellular oxalate flux is derived from anion exchange proteins belonging to the multifunctional SLC26 gene family. One of the gene family members, *Slc26a6*, is expressed at high levels in the intestine and proximal renal tubules and plays a major role in controlling systemic oxalate metabolism (Whittamore and Hatch, 2017). *Oxalobacter formigenes* produce a small protein that directly induces oxalate transport via the oxalate transporter SLC26A6–dependent mechanism in intestinal Caco-2 cells (Arvans et al., 2017).

Among oxalate transporters, many factors are involved in determining oxalate absorption and secretion in the gut in patients with ESRD: 1) dietary restriction; 2) high uremic toxin concentration; 3) malabsorption; 4) low blood concentrations of calcium, magnesium, and fiber that may affect the oxalate absorption; 5) use of antibiotics, phosphate binders, or other medications that can influence the quantitative and qualitative composition of gut microbiota; and 6) oxalate-degrading activity (ODA) of gut microbiota (Robijn et al., 2011; Mitchell et al., 2019; Ticinesi et al., 2020).

Gut microbiota is a critical factor affecting intestinal oxalate metabolism and kidney stone formation (Stepanova et al., 2018; Chen et al., 2019; Ticinesi et al., 2020). *O. formigenes* degrade oxalate in the intestine and stimulate its endogenous secretion (Hatch et al., 2006; Arvans et al., 2017). However, the absence of intestinal *O. formigenes* colonization may not be the only cause for kidney stones, and a diversity of gut ODB (e.g., *Lactobacillus spp., Bifidobacterium spp., Bacillus spp., E. faecalis, N. albigula*) were identified (Gomathi et al., 2014; Barnett et al., 2016; Miller et al., 2019; Stepanova et al., 2021). Since the gut microbiota in patients on dialysis is characterized by increased *Enterobacteriaceae* and low colonization of *Bifidobacterium* and *Lactobacillus* species compared with normal controls (Hu et al., 2020; Ren et al., 2020), the low abundance of ODB in gut microbiota may play a significant role in oxalate homeostasis in patients with ESRD. Indeed, we previously demonstrated that the ODB number in patients on dialysis was significantly lower than in healthy volunteers (Stepanova et al., 2020a; Stepanova et al., 2021). However, when we separately evaluated the ODB number and their total ODA in fecal microbiota in patients on dialysis, we got surprising results. The ODB number was associated neither with their total ODA in fecal microbiota nor urinary oxalate (UOx) excretion and POx concentration. According to the results, only total fecal ODA was associated with urine and plasma oxalate levels: the lower the ODA in the fecal microbiota, the higher the POx concentration and the lower the UOx excretion. Thus, total ODA in the fecal microbiota rather than the ODB number resulted in elevation of plasma and urine oxalate concentrations in patients with ESRD.

### Crosstalk Between Oxalate Homeostasis, Oxidative Stress, Inflammation, and CVD Risk in Patients With ESRD

**FIGURE 1:**
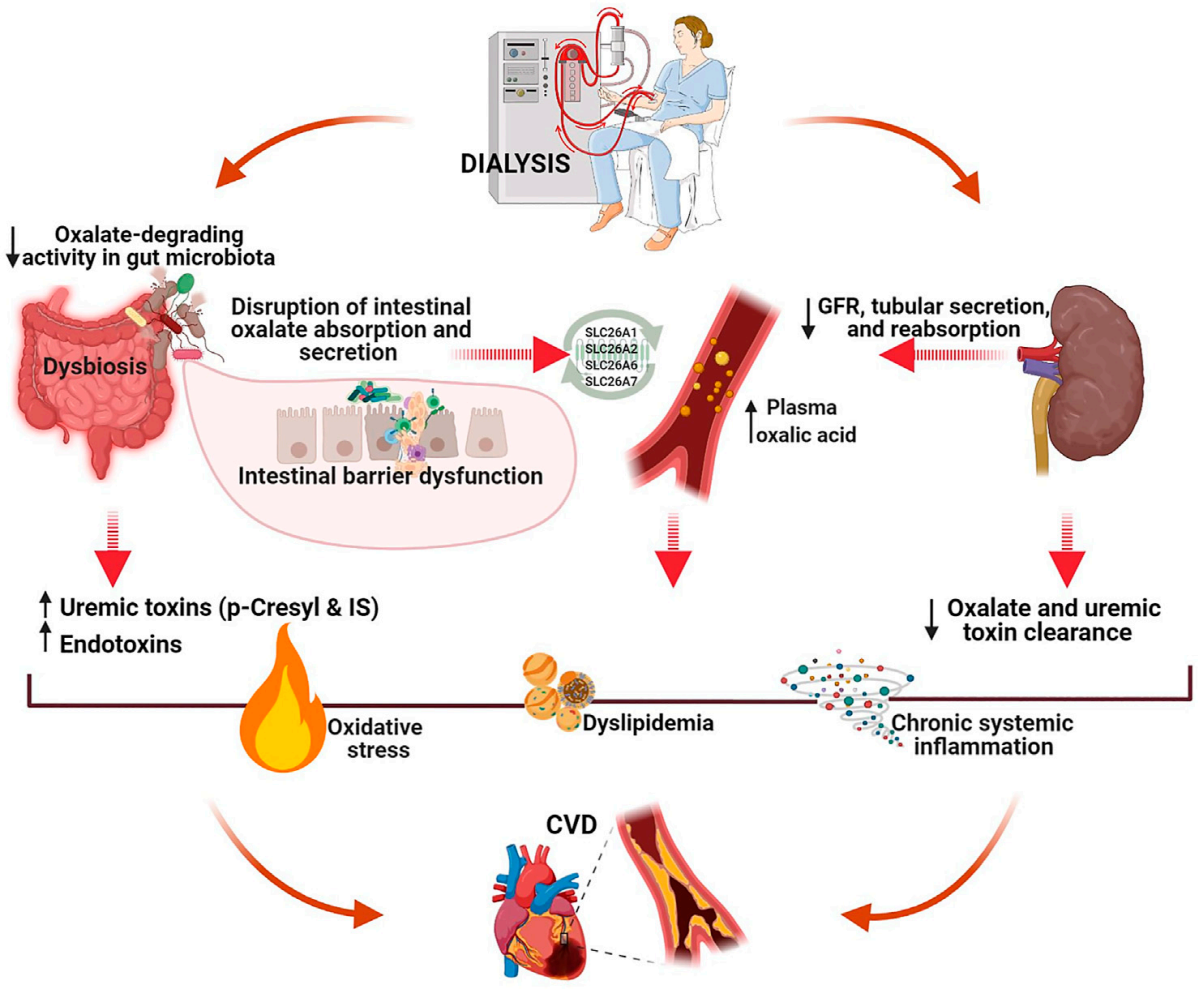
A proposed mechanism of the interaction between oxalate homeostasis and CVD in patients with end-stage renal disease (created with BioRender.com). Intestine plays a crucial role in oxalate homeostasis disruption in patients on dialysis. CKD-associated gut dysbiosis with commensal microbiota deficiency leads to decreased total fecal ODA, intestinal barrier dysfunction, and alteration of oxalate transport mechanisms in the intestinal epithelium (both secretion and absorption). Both uremia- and oxalate-induced molecular mechanisms initiated in the gut can activate oxidative stress, chronic inflammation, and atherogenesis, eventually resulting in CVD.

Patients with kidney stone disease have an increased CVD risk, probably due to the common origin of both diseases (Liu et al., 2014; Fan et al., 2017; Arafa et al., 2020). This common source is likely the gut microbiota (Cheungpasitporn et al., 2014; Ma and Li, 2018; Arafa et al., 2020; Zhou et al., 2020). Many mechanisms have been suggested to be involved in gut microbiota–mediated lipid metabolism disruption, including *Bifidobacteria-* and *Lactobacillus*-mediated fermentation of nondigestible carbohydrates, trimethylamine N-oxide-mediated impact of lipid absorption and cholesterol homeostasis, reduction of the total bile acids, oxidative stress initiation, immune cell recruitment, and differentiation (Ma and Li, 2018; Matey-Hernandez et al., 2018; Wolf and Ley, 2019; Zhou et al., 2020). Extrapolating the presented hypothesis to the relationship between impaired oxalate homeostasis and CVD in patients on dialysis, we believe that gut dysbiosis may lead to intestinal barrier dysfunction, disruption of oxalate transport mechanisms in the intestinal epithelium (both secretion and absorption), initiation of oxidative processes, chronic inflammation, and dyslipidemia. In line with the common origin hypothesis, our pilot study findings provide preliminary evidence that a decrease in total ODA in the fecal microbiota is associated with atherogenic dyslipidemia in patients with ESRD (Stepanova et al., 2020b). A proposed mechanism for the interaction between oxalate homeostasis and CVD in patients with ESRD is illustrated in Figure 1.

Oxidative stress and inflammation are major risk factors for accelerated atherosclerosis (Khan, 2014; Yang et al., 2017; Devarajan, 2018). However, excessive renal accumulation of CaOx crystals may also lead to the formation of reactive oxygen species (ROS) and the activation of oxidative processes and oxidative stress (Khan, 2004, 2014; Abhishek et al., 2017). ROS produced by activated NADPH oxidase due to oxalate dyshomeostasis oxidizes low-density lipoprotein (LDL) to minimally modified LDL (mm-LDL), which induces the secretion of various cytokines, including monocyte chemoattractant protein-1 (MCP-1) (Khan, 2004; Khan, 2014; Abhishek et al., 2017). MCP-1 recruits monocytes and transforms them into foam cells, owing to the modified lipoprotein (Khan, 2004; Khan, 2014; Abhishek et al., 2017). In addition to MCP-1, oxidatively modified LDL induces the synthesis of other proinflammatory cytokines, such as interleukin-6 (IL-6) and tumor necrosis factor-alpha (TNF-α) (Abhishek et al., 2017; Wolf and Ley, 2019). However, these mediators are strongly involved in oxalate-induced inflammation (Gaĭseniuk et al., 2013; Khan, 2014; Abhishek et al., 2017). CaOx can activate human monocytes, which increase TNF-α, IL-6, and MCP-1 production and lead to chronic inflammation (Dominguez-Gutierrez et al., 2018). In mirror to mentioned above, our recent study was the first to demonstrate the increasing trend in serum malondialdehyde and oxidative stress index according to the gradual increase in POx concentrations in patients with ESRD (Korol et al., 2021). Moreover, blood antioxidant balance has been associated with the POx concentration: the higher the POx concentration, the more pronounced the decreased levels of ceruloplasmin, SH-groups, and total peroxidase activity. In another study, we separately characterized the association of POx concentration with the lipid profile and proinflammatory markers in patients with ESRD, and identical results were obtained (Stepanova et al., 2020c). POx elevation was significantly associated with an increasing trend in the atherogenic lipoprotein fractions and proinflammatory markers and linearly decreased high-density lipoproteins. Moreover, POx concentration was directly correlated with blood triglycerides, IL-6, and MCP-1 levels and significantly associated with CVD event history during the 2-years follow-up period independently of other examined CVD risk factors (Stepanova et al., 2020c). Taken together, these findings have provided preliminary clinical evidence that elevated POx concentration is associated with increased levels of blood oxidative stress and proinflammatory markers and a high CVD risk in patients with ESRD.

On the other hand, oxidative stress and chronic inflammation, which are present in all CKD stages, with the highest levels in ESRD (Cobo et al., 2018; Vasylchenko et al., 2020), might themselves contribute to the reduced SLC26A6-mediated transcellular oxalate transport and the enhanced passive paracellular intestinal oxalate absorption (Amin et al., 2018; Bashir et al., 2019; Kumar et al., 2021). In an obese mouse model, oxidative stress and systemic/intestinal inflammation were found to reduce active intestinal oxalate secretion and increase passive intestinal oxalate absorption, eventually resulting in obesity-associated hyperoxaluria (Amin et al., 2018; Bashir et al., 2019). Therefore, this vicious circle of complex molecular mechanisms precludes the accurate determination of the root cause of oxalate dyshomeostasis and its relationship with CVD in patients with ESRD. Nevertheless, gut-origin oxidative stress, dyslipidemia, and chronic inflammation remain common links between impaired oxalate homeostasis and increased CVD risk.

### Final Considerations

In this opinion article, we present a new perspective on the pathophysiologic and translational relevance of impaired oxalate homeostasis in CVD development in patients with ESRD. To the best of our knowledge, although hyperoxalemia and CVD are well-known occurrences in ESRD, few studies have explored this issue. Collectively, we believe that in renal failure conditions, not only do a decrease in the secretion and increase in the reabsorption of oxalate in the kidneys lead to elevated POx but also does the intestine play a crucial role in the oxalate dyshomeostasis. CKD-associated gut dysbiosis with commensal microbiota deficiency leads to decreased total fecal ODA, intestinal barrier dysfunction, and alteration of oxalate transport mechanisms in the intestinal epithelium (both secretion and absorption). Both uremia- and oxalate-induced molecular mechanisms initiated in the gut can be simultaneously involved in the activation of oxidative stress, chronic inflammation, and atherogenesis. However, the role of gut microbiota in oxalate homeostasis and CVD is still far from being fully understood. Thus, we believe that our hypothesis would be reflected in further experimental and large-scale research to clarify the interaction mechanisms and the role of impaired oxalate homeostasis in CVD risk in patients with ESRD.

## Abbreviations

CKD, chronic kidney disease; CVD, cardiovascular disease; GFR, glomerular filtration rate; IS, indoxyl sulfate; ODA, oxalate-degrading activity.
